# Towards the Applicability of Measuring the Electrodermal Activity in the Context of Process Model Comprehension: Feasibility Study

**DOI:** 10.3390/s20164561

**Published:** 2020-08-14

**Authors:** Michael Winter, Rüdiger Pryss, Thomas Probst, Manfred Reichert

**Affiliations:** 1 Institute of Databases and Information Systems, Ulm University, 89081 Ulm, Germany; manfred.reichert@uni-ulm.de; 2 Institute of Clinical Epidemiology and Biometry, University of Würzburg, 97080 Würzburg, Germany; ruediger.pryss@uni-wuerzburg.de; 3 Department for Psychotherapy and Biopsychological Health, Danube University Krems, 3500 Krems, Austria; thomas.probst@donau-uni.ac.at

**Keywords:** process model, process model comprehension, electrodermal activity, sensor

## Abstract

Process model comprehension is essential in order to understand the five Ws (i.e., who, what, where, when, and why) pertaining to the processes of organizations. However, research in this context showed that a proper comprehension of process models often poses a challenge in practice. For this reason, a vast body of research exists studying the factors having an influence on process model comprehension. In order to point research towards a neuro-centric perspective in this context, the paper at hand evaluates the appropriateness of measuring the electrodermal activity (EDA) during the comprehension of process models. Therefore, a preliminary test run and a feasibility study were conducted relying on an EDA and physical activity sensor to record the EDA during process model comprehension. The insights obtained from the feasibility study demonstrated that process model comprehension leads to an increased activity in the EDA. Furthermore, EDA-related results indicated significantly that participants were confronted with a higher cognitive load during the comprehension of complex process models. In addition, the experiences and limitations we learned in measuring the EDA during the comprehension of process models are discussed in this paper. In conclusion, the feasibility study demonstrated that the measurement of the EDA could be an appropriate method to obtain new insights into process model comprehension.

## 1. Introduction

Process models are abstractions (i.e., in terms of documentation, definition, and execution) from the physical world representing objects, procedures, or issues [1]. Process models are used in particular to conceptualize, determine, and describe the procedures, technical systems, or the processes of organizations [2]. Regarding the latter, process models constitute a composition of activities, decisions, data, and resources from associated organizational processes in order to achieve a particular objective (e.g., product or service) [3]. However, process models do not solely document the processes of organizations; they additionally offer opportunities to extract specific process information (e.g., key performance indicators) for the purposes of process analysis, optimization, and automation [4]. In addition, process models provide a means for collaboration, thus facilitating the conveyance of process information among stakeholders [5].

In order to benefit from the application of process models, organizations must take care that the proper understanding of such models (i.e., process model comprehension) is ensured for all involved stakeholders [6]. Therefore, the identification of factors is vital, both positive and negative ones, which influence the comprehension of process models. For example, if factors that hinder process model comprehension are not addressed properly, the respective processes might not deliver the required results. Failures that happen in the application of such models have been commonly linked to model incomprehension [7].

In this context, a large body of research has evolved over the last decade on studying the factors that influence the comprehension of process models [8]. Therefore, different objective properties of process models, such as the syntax [9], structure [10], labeling [11], coloring [12], visual notational deficiencies [13], and secondary notation [14] were investigated, their influence on process model comprehension elaborated, and corrective actions presented (e.g., guidelines [15]).

However, existing research in the context of process model comprehension addresses only objective factors. Since different kinds of stakeholders (e.g., modeling and domain experts) are involved in working with process models, their expertise in the comprehension of such models varies [16]. Interestingly, research showed that expertise in working with process models is not the only decisive factor influencing process model comprehension [17]. Moreover, despite existing research in the context of process model comprehension, stakeholders, both experienced and inexperienced, are still facing challenges on how to properly read and comprehend process models. Therefore, the influence of subjective factors and their influence on model comprehension was addressed in recent works. Examples are the modeling expertise [18], model reader preferences [19], learning strategies [20], perceptual discrimination [21], or perceived usefulness [22].

As is known from other domains, focus is increasingly put on the influence of cognitive aspects [23]. For example, in the context of process model comprehension, the mental load as well as the corresponding efforts [24,25], cognitive style [26], cognitive biases [27], and cognitive load [28] are investigated. Moreover, additional technologies are applied to get a deeper understanding of the cognitive aspects and their influence on the comprehension of process models. Prominently, the application of eye tracking (e.g., [29,30,31,32]) is pursued. Additionally, the use of other technologies and methods (e.g., smart mobile devices [33], serious games [34]) are becoming more popular in this context.

As such technologies (e.g., eye tracking [35]) become increasingly affordable and are fanned by the proliferation of additional sensors (e.g., in smart mobile devices [36]), the identification of factors influencing process model comprehension can thus be facilitated for research in a novel manner. More specifically, the application of smart sensors allows for the analysis of factors not previously considered in the context of process model comprehension. Examples could be the measurement of physiological parameters (e.g., heart rate [37]) or psychological factors such as the level of arousal [38]. Regarding the latter, research showed that the level of arousal and the different states (e.g., tension, excitement) thereof affect our behavior (e.g., decision making [39]). Usually, the level of arousal (i.e., the state of being awake and attentive) is measured either by self-reporting tools (e.g., questionnaires [40]) or through the application of technologies (e.g., wearable sensors [41]) that measure body reactions against environmental influences. Other examples are the consideration of pupil dilation with eye tracking [42] or brain activity with the electroencephalography (EEG) [43]. Another approach for the analysis of the level of arousal is the measurement of the skin conductivity, also known as the electrodermal activity (EDA) [44,45,46].

For this reason, to the best of our knowledge, no works exist that considers the electrodermal activity (EDA) in the context of process model comprehension. Therefore, this paper presents first insights, experiences, and lessons learned gathered in EDA research (i.e., preliminary test run and feasibility study) during the comprehension of process models. The emphasis is put on the research question, which evaluates the applicability as well as the appropriateness of measuring the EDA relying on a smart EDA and physical activity sensor during the comprehension of process models. Moreover, the work at hand shall, on the one hand, foster further EDA studies in this context and, on the other hand, shall contribute towards research to facilitate an in-depth neuro-centric perspective in terms of process model comprehension.

The remainder of this paper is structured as follows: Section 2 introduces the theoretical background about the EDA. In addition, this section describes the study context and the setting of the conducted EDA research (i.e., test run and feasibility study). The obtained EDA results are presented, tested for significance, and discussed in Section 3. Moreover, this section presents the limitations and lessons we learned. Finally, Section 4 summarizes the paper and gives an outlook on future work.

## 2. Materials and Methods

### 2.1. Electrodermal Activity

**Figure 1:**
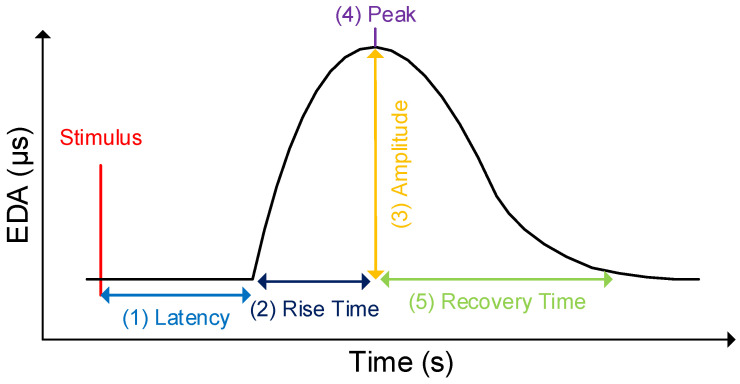
Skin conductance response (SCR) after a stimulus.

The electrodermal activity (EDA) describes variations in the eccrine sweat gland production of the human body. These variations in the sweat production result in changes of the electrical skin properties (i.e., skin conductance) [47]. Notably, sweating is controlled by the sympathetic nervous system (i.e., part of the autonomic nervous system), and changes in the skin conductance are indications of physiological or psychological arousal (e.g., fight-or-flight response) [48]. Research demonstrated that this kind of arousal is significantly related to brain functions that regulate motor, sensory, and cognitive skills [44,49,50]. For example, when emotionally agitated (e.g., on the eve of an exam), sweat production is increased, resulting in an increase in the EDA as well (e.g., higher cognitive load). In turn, at rest, sweat production and the associated EDA is low. In general, the EDA is measured with the application of sensors that are attached either on the sole of the feet or the palms. The reason for this way of attachment is that the number of eccrine sweat glands is highest in these two places. Furthermore, the EDA describes a raw electric signal that consists of two components characterizing the phasic skin conductance response (SCR) and the tonic skin conductance level (SCL) [51]. More specifically, the SCR constitutes abrupt increases in the skin conductance as a direct reaction to an environmental stimulus. Usually, these abrupt increases emerge between 1 and 5 s after the presentation of a stimulus (e.g., picture, sound). Such increases are strongly associated with cognitive processes (e.g., decision making) following a short-term event [52]. The SCR (specified in microsiemens, μs) is characterized by five factors over time after the appearance of a stimulus: (1) latency, (2) rise time, (3) amplitude, (4) peak, and (5) recovery time. While the latency has a usual duration between 1 and 5 seconds, the duration of the other four factors is dependent on the individual, as well as the presented stimulus. An example of the SCR is depicted in Figure 1. In addition, SCR can occur spontaneously in the absence of any stimulus (i.e., non-SCR) [47].

**Figure 2:**
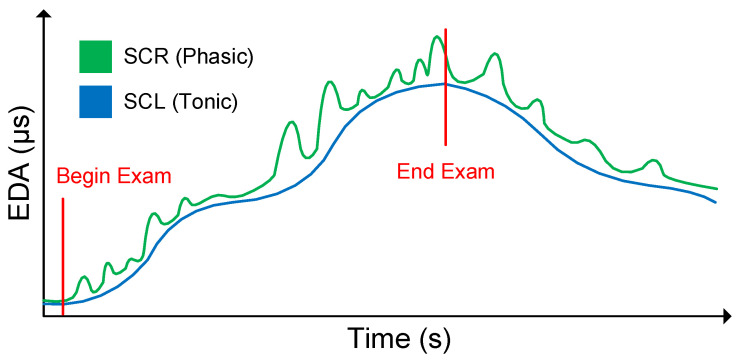
Distinction of the tonic skin conductance level (SCL) and phasic skin conductance response (SCR) in a raw EDA signal.

In contrast to phasic SCR, the tonic SCL (specified in microsiemens, μs) is defined as the slowly changing raw level of skin conductance. Changes in the SCL are not triggered by particular stimuli or events, but represent a continuous intra-individual course over the period of time. The SCL varies significantly between individuals and is affected by psychological states, physical condition, and autonomic regulation. Moreover, the size of the electrodes used for measuring the EDA signal is an additional influencing factor. Although the phasic SCR is more prominent in EDA research, insights revealed the importance of considering both components (i.e., SCR and SCL) in order to better understand the physiological as well as the psychological processes and their reactions to specific stimuli [53]. In this context, Figure 2 exemplarily outlines the distinction between SCR (i.e., phasic component) and SCL (i.e., tonic component) in a raw EDA signal while writing an exam. The exam situation leads to a slow and continuous increase in the SCL (i.e., blue), since the exam represents a tense situation requiring an attentive state. While solving single tasks in the exam, there are repeatedly abrupt increases in the SCR (i.e., green) over time. These abrupt increases are indications of the fast and short-term amplifications of cognitive processes (e.g., reasoning, decision making). When the exam has passed, and the caused tension declines, the SCL also decreases steadily towards a baseline level.

### 2.2. Context Selection

In general, comprehension is a cognitive process that is strongly affected by the level of arousal having an impact on, for example, reading [54], learning [55], and information processing [56]. Similarly, the comprehension of process models is a complex matter. On the one hand, there must be an adequate level of knowledge about the process modeling notation used for the creation of the respective process models. On the other hand, documented information in process models needs to be decoded and captured properly by all stakeholders [17]. Existing research has already made major contributions in the context of process model comprehension. However, the measurement of the EDA has not been addressed so far in prior works. To address this gap, the paper at hand investigates the following research question:


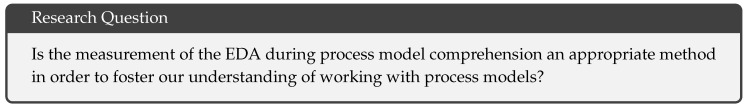


This paper presents the first insights into measuring the EDA in the context of process model comprehension. The insights obtained shall contribute to a novel neuro-centric perspective for research on the comprehension of process models. Our existing conceptual framework for the comprehension of process models that already incorporates methods and theories from cognitive neuroscience and psychology is therefore enriched by the findings from this work [57]. A preliminary test run and a feasibility study were conducted relying on the measurement of the EDA with a specific EDA and physical activity sensor. The purpose of the test run was to familiarize ourselves with the measurement of the EDA. This included the application of the measuring sensor device, execution of the EDA measurement, as well as the analysis and interpretation of the results. With the knowledge gained from the test run, a feasibility study was conducted, in which participants needed to comprehend a set of differently complex process models, while, at the same time, their EDA was recorded.

### 2.3. Instrumentation

**Figure 3:**
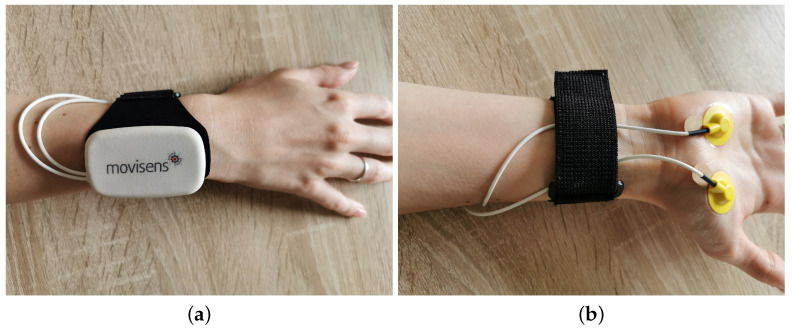
Attachment of the sensor EdaMove 3. (**a**) Top side of the EdaMove 3 sensor; (**b**) Electrodes attached to the palm.

For both phases (i.e., test run and feasibility study), the EDA and physical activity sensor EdaMove 3 was used [58]. The EdaMove 3 is a smart sensor device to record and analyze skin conductivity (i.e., EDA), as well as physical activity (e.g., step counter). The sensor has already been successfully applied in different fields of research (e.g., psycho-physiologic monitoring [59], affective computing [60]) and provides all relevant standards for the proper measurement of the EDA and corresponding components (i.e., phasic SCR, tonic SCL). In more detail, an exosomatic measurement with direct current (DC) (i.e., 0.5 V constant voltage system) is used for the assessment of the EDA signal. The measurement range is between 0 μs and 100 μs, and the accuracy is <1.5%, with a resolution of 14 bit. Additionally, the sensor enables a live analysis of the EDA, body temperature, step count, and movement acceleration in all dimensions. The EdaMove 3 has two reusable electrodes (i.e., non-polarizing sintered Ag/AgCl) with an electrode disc diameter of 10 mm (i.e., 78.5 mm^2^ effective electrode area). The electrodes can be attached either on the sole of the feet or the palms with adhesive tape rings supported by an electrode gel and alcohol in order to measure the EDA with a sampling rate of 32 Hz (bandwidth: DC to 8 Hz). In the presented research, the sensor was attached to the palms (see Figure 3a,b). The EdaMove 3 was configured with all relevant information (e.g., time for the start of the measurement) with the Movisens SensorManager 1.14.4. Recorded sensor data were visualized and preprocessed for the first analyses using the UnisensViewer 1.12.38. Furthermore, DataAnalyzer 1.11.12 was used for data transformation and the calculation of relevant measures (e.g., SCL, SCR). Note that the detection parameters of DataAnalyzer were based on the proposed approaches in [53,61]. More specifically, the tonic component of the EDA signal (i.e., SCL) was low pass filtered (i.e., second order Butterworth) with a filter frequency of 0.1 Hz. Phasic SCRs were detected automatically (i.e., second order high pass Butterworth filter with 0.1 Hz) with the consideration of the following criteria: (1) default minimal rise time for SCR detection > 0.05 μs/s, (2) default minimal amplitude for SCR detection > 0.1 μs, and (3) default maximal rise time for SCR detection < 0.9 s. Importantly, there is an exception in SCR peak detection, which occurs with overlapping (i.e., superimposed) peaks. More specifically, instead of a signal drop after reaching the peak (i.e., recovery time), an ascent of the signal caused by another SCR peak is detected. In this special case, the first emerging peak can be determined with an amplitude > 0.05 μs [61]. In general, the parameters, which constituted a potential limitation (see Section 3.3), were defined by the vendor of EdaMove 3 as a reasonable compromise in order to allow an offline as well as online live analysis in this context. Further, the stimuli (i.e., pictures in the test run, process models in the feasibility study (see Section 2.4 and Section 2.5) were presented on a 23” monitor (resolution of 1920 × 1080, 96 PPI). Finally, SPSS 26 was used for all statistical analyses.

### 2.4. Preliminary Test Run

**Figure 4:**
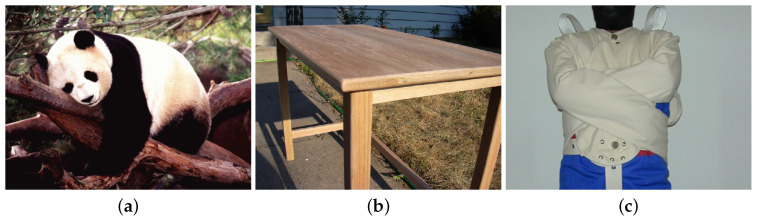
Pictures from the Geneva Affective Picture Database (GAPED). (**a**) Positive: giant panda; (**b**) neutral: table; (**c**) negative: straitjacket.

**Figure 5:**
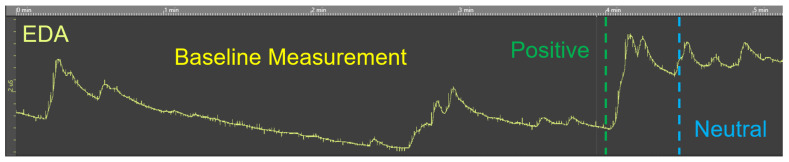
Excerpt from an EDA measurement.

The purpose of the small-scale preliminary test run was to familiarize ourselves with the measurement of the EDA. This included the accurate application of the EdaMove 3, correct measurement of the EDA signal as well as the respective components (i.e., SCL, SCR), and the correct analyses of the recorded EDA data. Therefore, n = 4 participants had to contemplate various pictures obtained from the Geneva Affective Picture Database (GAPED) [62]. Note that all participants gave their informed consent for inclusion before they participated in the test run. GAPED contains a total of 730 pictures in terms of emotion induction. The pictures represent either humans, animals, or objects that are related to positive, negative, or neutral emotions. These pictures were rated regarding arousal, valence, and accordance with internal and external norms. Figure 4 exemplarily presents pictures that should arouse positive (i.e., (a) giant panda), neutral (i.e., (b) table), or negative (i.e., (c) straitjacket) emotions. In order to improve our experience with the EdaMove 3 and the measurement of the EDA, the pictures from GAPED were shown to the participants in short trials with different settings. For example, the display duration of the pictures varied (from 1 up to 15 s); the periods of rest in order to identify a proper baseline level to start with the measurement were between 1 and 15 min; and participants needed to indicate whether a picture triggered positive, negative, or neutral emotions. Figure 5 presents an excerpt of the EDA signal measured with EdaMove 3 (i.e., UnisensViewer). The figure depicts the raw EDA signal (i.e., no distinction between tonic SCL and phasic SCR) showing the baseline measurement (i.e., from Minute 0 to 4) as well as the presentation of a positive (i.e., from Minute 4) and neutral (i.e., from Minute 4.30) picture from GAPED. Generally, the EDA findings that we gained from the test run were in accordance with existing literature (e.g., the EDA was higher in positive-related pictures compared to neutral ones) [47,63,64]. Furthermore, the experiences we gathered and the lessons we learned from the test run are included in the discussion presented in Section 3.4.

### 2.5. Feasibility Study

For the investigation of the research question (see Section 2.2), using the gained experiences from the test run, a feasibility study was conducted, which was performed as follows:

Participants: The feasibility study included a total of n = 9 participants. All participants were students (i.e., 2 were female; all participants were under 25 years), and the study was conducted at Ulm University. Prior to the feasibility study, all participants gave their informed consent for inclusion. The participants were divided into two groups (i.e., Groups A and B) using the round-robin approach (i.e., alternating assignment to Group A or B). Group A consisted of n = 5 and Group B of n = 4 participants.

**Figure 6:**
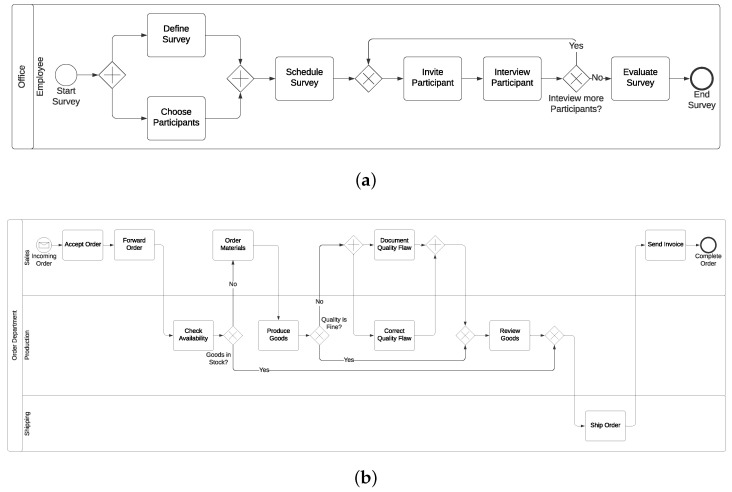
Used process models in the feasibility study. (**a**) Easy process model: implementation of a survey; (**b**) complex process model: order for goods.

Materials: Two differently complex process models (i.e., easy and complex) expressed in terms of the Business Process Model and Notation (BPMN) 2.0 were used in this study [65]. The process models were composed of basic elements of BPMN 2.0. Note that the easiest model showed the implementation of a survey. In turn, an order for goods was presented in the complex one. Figure 6 shows both process models: Figure 6a presents the survey scenario, while Figure 6b the order for goods (high-resolution images of the used process models are available here: https://tinyurl.com/y7g5hgy2). More specifically, in order to ensure differences in process model complexity, the two models were created based on the metrics proposed in [66]. Therefore, the two process models varied in the number of modeling elements and outgoing sequence flows. The following Table 1 presents the number of process modeling elements, each used in the two respective models.

Measures: Although various parameters (e.g., SCR energy, recovery time) can be considered in EDA analyses [47], for the first evaluation of the applicability of the EDA in the context of process model comprehension, the following three EDA-related measures were considered in the feasibility study:

- Mean SCL: The tonic SCL describes the changing level of skin conductivity over a period of time (see Section 2.1). A state of physiological or psychological arousal usually leads to variations (e.g., increase) in the SCL. In our context, the comprehension of process models constituted a cognitively challenging task that created a state of arousal (e.g., attentive). Therefore, the analysis of the SCL allowed for the assumption about whether the comprehension of differently complex process models results in variations (e.g., elevation) in the SCL.
- Number of SCR peaks: SCR peaks are parts of the phasic component that are indications of short-term processes with high physiological (e.g., the wait for the go-ahead) or psychological (e.g., decision making) demands. In process model comprehension, the correct interpretation of process information must be ensured and, therefore, decisions (e.g., which activities may run in parallel) must be made, which are decisive for the perception as well as the correct comprehension of the process model. For this reason, it was interesting to evaluate whether the number of SCR peaks was higher in the complex process model juxtaposed with the easy model. Importantly, only SCR peaks with an amplitude height of >0.1 μs were considered (i.e., special case with >0.05 μs; see Section 2.3).
- Mean of SCR amplitudes energy level: The mean of SCR amplitudes energy level is a measure in order to record the degree of stress (e.g., cognitive load) a stimulus or event provokes. The higher perceived stress is, the higher is the amplitude and vice versa. In our study, the evaluation of the SCR amplitudes revealed insights about the cognitive load and related processes during the comprehension of process models.

**Figure 7:**
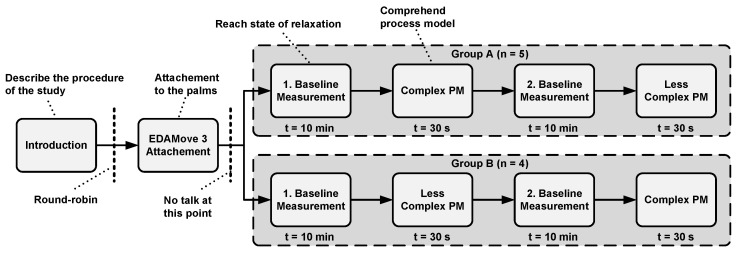
Study design used in the feasibility study.

Study design: The design of the feasibility study was based on the guidelines proposed in [67]. The study was conducted in a prepared lab at Ulm University. The lab was quiet and ambiently dimmed, and care was taken to keep the lab temperature around 22 degrees Celsius. Such preparations were necessary in order to ensure the same study setting, since environmental influences (e.g., temperature) have an impact on the EDA. The experiences we gained from the preliminary test run (see Section 2.4) contributed to the study design. Due to the availability of only one sensor device, only one participant could be evaluated, and each study session took about 25 min. A study session was as follows: The participant was welcomed, and the study procedure was explained. Afterwards, informed consent as well as demographic information were provided. Following this, participants were assigned to Group A or B using the round-robin approach (i.e., alternating assignment to Group A or B) in order to ensure a balanced distribution in both groups across all participants. Group A started with the comprehension of the easy process model and then the complex one. In turn, Group B first had to comprehend the complex model, followed by the easy one. Then, the EdaMove 3 was attached to the palm (see Section 2.2). After the attachment of the sensor device, the participant was asked not to talk for the remaining duration of the study. After completing all these steps, a first baseline measurement was made. The participant was advised to sit comfortably, remain calm, and relax for a total of 10 minutes. Research has shown that in a state of relaxation, the EDA drops towards a baseline, from which the EDA measurements can be started [47,51]. Such arrangements (e.g., not to talk, relaxation) are necessary in order to avoid potential external influences, which may have an impact on the EDA signal. After 10 minutes, either the complex (i.e., Group B) or the easy (i.e., Group A) process model was shown to the participant for a total of 30 s. In these 30 s, the participant should comprehend the presented process model syntactically, as well as semantically. Subsequently, after comprehending the first model, a second baseline measurement was made in order to ensure that the EDA dropped towards the baseline level again. After the second baseline measurement, the second process model (i.e., either the complex (i.e., Group A) or easy one (i.e., Group B)) was shown for comprehension for another 30 s. In total, one EDA measurement was obtained from each participant that was divided into four parts: (1) Baseline Measurement 1, (2) Baseline Measurement 2, (3) comprehension of the easy process model, and (4) comprehension of the complex process model (see Section 3). Finally, Figure 7 summarizes the study design.

## 3. Results

**Figure 8:**
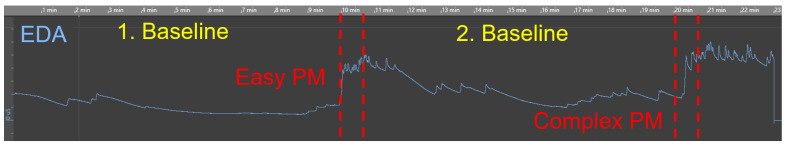
Presentation of a raw EDA signal.

Figure 8 shows the recording of a raw EDA signal measurement (i.e., without a separation of the tonic and phasic EDA components SCL and SCR) from a participant of Group A. In more detail, the figure depicts the two baseline measurements, as well as the presentation of the easy and the complex process model (PM). It can be seen from the figure how the EDA signal reaches a baseline within the first 10 minutes and has a clear burst increase after presenting the first process model for the purpose of comprehension to the participant. Afterwards, in the second baseline measurement, the EDA signal is a little bit more unsettled juxtaposed with the EDA signal in the first baseline measurement. A reason for this behavior of the EDA signal might be the level of arousal, which is the physiological and psychological state responsible for different behavioral and cognitive processes, such as attention, decision making, and information processing. It can be assumed that after the comprehension of the first process model, the participant may have been busy recapitulating and processing the process information just presented in the first model.

**Figure 9:**
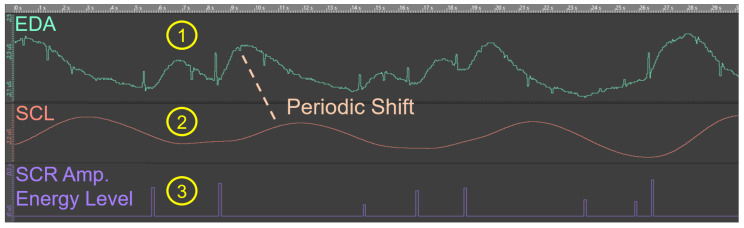
Analysis of the EDA measures: (1) EDA signal, (2) SCL, and (3) SCR amplitudes energy level.

From Figure 8, the tonic part of the EDA signal (i.e., SCL) is clearly visible. However, the distinction of the phasic part (i.e., SCR peaks) is hardly possible from this observation. For this reason, the two EDA components (i.e., SCL and SCR peaks) were considered separately by applying data transformation (i.e., signal decomposition) provided by the application DataAnalyzer (see Section 2.3). Note that the visualization of the phasic SCR component is currently limited in DataAnalyzer (see Section 3.3). Figure 9 presents exemplarily the considered measures from the feasibility study (see Section 2.5) after data transformation. The (1) raw EDA signal is split into the (2) tonic SCL and (3) phasic SCR amplitudes energy level. Regarding the latter, corresponding SCR peaks (i.e., 8 in total) and the related energy level (in μs) are well recognizable and visualized as abrupt increases in this figure. Note the small periodic shift of about 1 second in (2) SCL that was caused due to filtering. In more detail, the SCL was extracted from the raw EDA signal by means of a second order Butterworth filter (i.e., the output signal is shifted in time with respect to the input signal). In order to address a signal delay, further signal processing methods (e.g., IIR filter) should be applied (see Section 3.3).

The following tables present the results obtained from the feasibility study for the easy (see Table 2) and the complex (see Table 3) process model. For each participant (P) in the respective group (i.e., Group A or B), the mean and standard deviation of the three considered measures (M; see Section 2.5), mean SCL (in μs), number of SCR peaks, and mean of SCR amplitudes energy level (in μs) are shown in the tables. Further, only SCR amplitudes exceeding the threshold of >0.1 μs are considered (i.e., special case with >0.05 μs; see Section 2.3). Moreover, the averages for all measures obtained from all participants for the respective group are shown in both tables.

Moreover, Table 4, Table 5 and Table 6 present the mean and standard deviation (i.e., M (SD)) of the three obtained measures (see Section 2.5) during the two baseline measurements. More specifically, in Table 4, for each process model (i.e., easy and complex), the mean SCL (in μs) obtained in the respective baseline measurement (BM) from each participant (P) of Groups A and B before the comprehension of the respective model is shown, as well as the aggregated results of all participants from the respective group. In Table 5, for each 30 s time interval during the baseline measurements, the number of non-SCR peaks (i.e., absence of stimulus) from all participants and the aggregated results are shown. Finally, Table 6 depicts the mean of SCR amplitudes energy level (in μs, with threshold >0.1 μs, special case with >0.05 μs) for each participant and the average of all participants.

**Figure 10:**
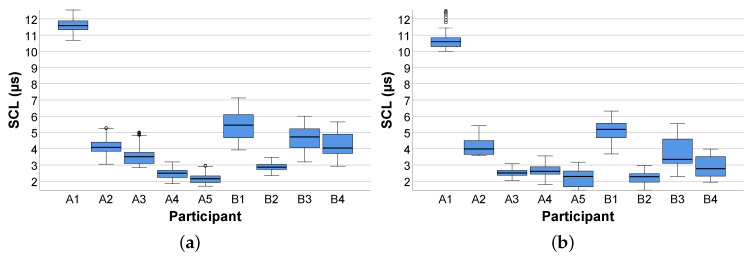
Skin conductance level during baseline measurements before process model comprehension. (**a**) Easy process model; (**b**) complex process model.

**Figure 11:**
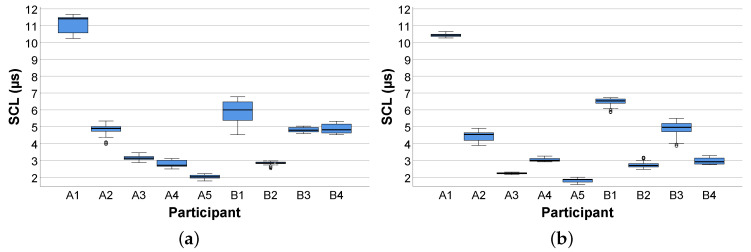
Skin conductance level during process model comprehension. (**a**) Easy process model; (**b**) complex process model.

In general, the results we obtained from the feasibility study were in line with results from the EDA research in different fields [47,68,69]. More specifically, during the baseline measurements and the comprehension of the process models, inter- and intra-individual variations in the mean SCL were encountered. The box plots shown in Figure 10 and Figure 11 demonstrate these SCL variations (in μs). In more detail, the box plots in Figure 10 show the SCL obtained from the participants during the baseline measurements (see Section 2.5) before the comprehension of the easy (see Figure 10a) and complex (see Figure 10b) process model. In turn, Figure 11 presents the SCL from the participants during the comprehension of the easy (see Figure 11a) and complex (see Figure 11b) process model. Regarding the inter-individual variations, the SCL in the participants reflected distinct differences in the skin conductivity (see Section 2.1) during the baseline measurements, as well as the comprehension of the process models. In terms of inter-individual variations, participants showed differences (e.g., general elevation in the SCL during the comprehension of the process models) in their respective SCL during the measurement of the baseline, as well as in model comprehension.

Considering the number of SCR peaks (i.e., phasic component), the results (see Table 2 and Table 3) showed that more SCR peaks appeared during the comprehension of the complex process model in both groups juxtaposed with the easy model. Hence, the results indicated a higher cognitive load in related cognitive processes (e.g., reasoning, decision making) during the comprehension of the complex process model. Moreover, considering Groups A and B, their results regarding the SCR peaks were similar for the easy process model. However, regarding the complex model, the individual results varied. The number of SCR peaks was higher in Group B compared to Group A. An explanation might be that participants from Group A, who saw the easy process model first, were already prepared to comprehend the second complex model. In turn, Group B needed to comprehend the complex process model first and, consequently, were subjected to more cognitive stress in order to comprehend the first model properly.

In the context of the SCR amplitudes energy level (see Table 2 and Table 3), except for Group B in the easy process model, the amplitudes were at an average energy level of about 0.30 μs. Research demonstrated that the average amplitudes energy level varies between 0.20 μs and 0.60 μs, which are references for various cognitive processes (e.g., reasoning) [70].

In the context of the two baseline measurements (see Table 4, Table 5 and Table 6), the obtained results assumed similar values for the three considered measures. Moreover, similarities in the mean SCL and mean SCR amplitudes energy level with the results obtained during the comprehension of the process models (see Table 2 and Table 3) were discernible. Regarding the number of SCR peaks in the easy process model, the average number was slightly higher during process model comprehension compared to the baseline measurements. Furthermore, in the complex process model, the number of SCR peaks differed notably (i.e., higher during model comprehension) juxtaposed with the number in the baseline measurements. As a result, it appeared that the number of SCR peaks was the only measure indicating distinctions (i.e., inter-individual) during the comprehension of differently complex process models.

Finally, Table 7 presents the aggregated results (i.e., mean and standard deviation) for the easy and complex process model regarding the three considered EDA measures (i.e., mean SCL (in μs), number of SCR peaks, mean SCR amplitudes energy level (in μs); see Section 2.5) from the participants of Groups A and B.

Regarding the mean SCL during process model comprehension and the SCR amplitudes energy level, only small differences can be seen (see Table 7). However, it appears that the number of SCR peaks was higher during the comprehension of the complex process model juxtaposed with the easy one. As with previous observations (see Table 2, Table 3, Table 4, Table 5 and Table 6), the results for the three measures in Table 7 reflected similar average values, but the number of SCR peaks in the complex process model further confirmed the indication as a measure in order to observe distinctions during process model comprehension of varying model complexity.

### 3.1. Inferential Statistics

To evaluate whether the differences between the easy and complex process models seen in the descriptive results (see Table 7) reached statistical significance, the Wilcoxon signed-rank test for two related samples was performed for the three considered EDA measures (i.e., mean SCL, number of SCR peaks, mean of SCR amplitudes energy level; see Section 2.5). All statistical tests were performed two-tailed, and the significance value was set to *p* < 0.05. Additionally, for each measure in the respective process model, the mean (M), standard deviation, and median (Mdn) are reported.

- Mean SCL: The Wilcoxon signed-rank test indicated that the mean SCL in the complex process model (M = 4.34 (2.71), Mdn = 3.04) was not significantly higher than the mean SCL in the easy process model (M = 4.34 (2.72), Mdn = 4.82), Z=−1.362,p<0.173.
- Number of SCR peaks: The Wilcoxon signed-rank test indicated that the number of SCR peaks in the complex process model (M = 7.00 (1.94, Mdn = 7.00)) was significantly higher than the number of SCR peaks in the easy process model (M = 4.89 (1.27), Mdn = 5.00), Z=−1.975,p<0.048.
- Mean of SCR amplitudes energy level: The Wilcoxon signed-rank test indicated that the mean of SCR amplitudes energy level in the complex process model (M = 0.29 (0.12), Mdn = 0.29) was not significantly higher than the mean of SCR amplitudes energy level in the easy process model (M = 0.34 (0.23), Mdn = 0.23), Z=−0.059,p<0.953.

In conclusion, the differences in the mean SCL between Groups A and B was not significant. Reasons could have been, on the one hand, the large disparity in the SCL between participants (i.e., inter-individual), which is associated with the application of such measurement (see Section 2.1), and, on the other hand, the equally distributed heterogeneity of intra-individual SCL variations in both groups. However, the number of SCR peaks was significantly different, and more SCR peaks occurred in the comprehension of the complex process model, which implied an agitated level of arousal (e.g., higher cognitive load) compared to the easy model. Finally, the mean of SCR amplitudes energy level showed no significant differences, which indicated that the participants were concerned with the correct comprehension of the respective process models. Summarizing, inferential statistics confirmed that the number of SCR peaks was the only measure in the feasibility study determining distinctions during the comprehension of differently complex process model.

### 3.2. Discussion

**Figure 12:**
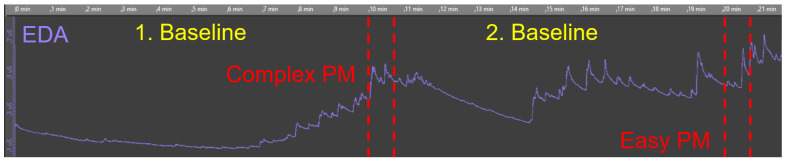
Challenges in analyzing the EDA signal

The focus of this work was to answer the research question (see Section 2.2) about whether the measurement of the EDA relying on a smart sensor (i.e., EdaMove 3) during process model comprehension is an appropriate method to foster our general understanding of working with such models. The results obtained from the feasibility study (see Section 3) indicated that participants from both groups (i.e., Groups A and B) were in a higher state of cognitive arousal during the comprehension of the process models. Moreover, the results regarding the tonic SCL revealed why in most research only the phasic SCR is more prominent (see Figure 10 and Figure 11) [47]. Since changes in the SCL are due to different factors and may vary inter- and intra-individual (see Section 2.1), their interpretation is often difficult. For example, whether a mean SCL of 6 is low, average, or high depends on the individual (similar for the SCR) [53]. In our context, similar to the work presented in [71], it could be shown that the comprehension of process models stimulated the level of arousal, which consequently led to noticeable variations (i.e., inter- and intra-individual) in the SCL from each participant over a period of time. However, especially inter-individual variations were consistent when observed repeatedly over a longer period of time. In the context of intra-individual variations, since all participants were not engaged in any other activity than comprehending the process models, the variations obtained during the comprehension of the easy and complex process model were alike. In general, during process model comprehension, not only the semantic process information must be comprehended properly, but it must be also ensured that the syntactic information of the respective process modeling notation (i.e., BPMN 2.0) is correctly comprehended. In this context, comprehension is a psychological process, which can be measured with the consideration of the EDA [72]. More specifically, comprehension leads to an increase in the EDA due to higher activation in the eccrine sweat gland production. Moreover, the appearance of significantly more phasic SCR peaks (see Section 3.1) in the complex process model revealed that participants were under higher cognitive load during model comprehension. The reason is that in the complex process model (see Figure 6b) more information must be interpreted and comprehended correctly compared to the easy model (see Figure 6a). Furthermore, this measure (i.e., number of SCR peaks) was identified in the feasibility study as the only measure that was able to recognize significant distinctions during the comprehension of the differently complex process model (i.e., easy and complex). The obtained SCR amplitudes energy levels indicated that participants (i.e., inter- and intra-individual) were mainly occupied with the comprehension of the presented process models and the related cognitive process (e.g., reasoning, decision making) [70]. Higher SCR amplitudes energy levels are particularly evident in aversive situations (e.g., fear) or during heavy physical exertion (e.g., weightlifting). In addition, comparing the three measures during process model comprehension with the ones obtained from the two baseline measurements, the latter showed minimal differences (i.e., lower values). However, the results confirmed the indication that the number of SCR peaks was the only significant measure in the feasibility study in order to measure process model complexity. In general, our results in the context of process model comprehension were in line with related EDA results obtained in other studies with different emphases [47,53,73,74,75]. Moreover, similar to the correlation between the EDA and the difficulty in tasks shown in the work of [69], there might be a correlation between EDA and process model comprehension resulting in a correlated elevation of the EDA with increasingly complex process models (see Section 4). While in single short-term observations within seconds, the interpretation of the emergence of a SCR peak can be attributed to the cause (e.g., presentation of an external stimulus). However, in long-term observations, such an attribution only by measuring the EDA is difficult. Referring to Figure 9, a total of eight SCR peaks are shown during the 30 s presentation of the process model. The reasons for the appearance of the SCR peaks could have been manifold (e.g., classification or revision of comprehended process information), but in the end, only assumptions about their appearance can be made. An approach allowing for a better interpretation and better supporting the causality would be the collection of more information (e.g., self-reporting tools) and further parameters with additional technologies (e.g., smart sensors) during the measurement of the EDA [63]. For example, the recording of eye movements may assist in a better interpretation of the SCR peaks. With the correlation of the eye movements at the time of an SCR peak, more concrete interpretations about the reasons for the emergence of an SCR peak can be derived. Moreover, another challenge that needed to be considered during the EDA measurement is demonstrated in Figure 12. More specifically, the figure visualizes the raw EDA signal of a participant from Group B. At first, similar to Figure 8, the first baseline measurement leads to a stabilization of the EDA signal towards a baseline level. However, from minute 6 on in the first measurement and from minute 14 on in the second one, a significant and unsettled increase in the tonic (i.e., SCL) as well as in the phasic (i.e., SCR) EDA signal were recognizable. Especially in the second baseline measurement, the peaks were evident, although, as with the other participants in the feasibility study, the study setting was the same. However, it can only be conjectured what the possible reasons were (e.g., external influences). Consequently, it is important to keep an eye on such variations as they may affect the actual EDA measurement.

Summarizing, we demonstrated successfully in a feasibility study that the measurement of the EDA with the application of a specific EDA and physical activity sensor (i.e., EdaMove 3) during process model comprehension is an appropriate method in order to foster research in this context. Although there were several limitations and aspects that need to be considered carefully (see Section 3.3 and Section 3.4), the measurement of the two EDA components (i.e., tonic SCL and phasic SCR) provides interesting insights to support our understanding of working with such models. Research as well as practice may benefit both in the future from the obtained insights. For example, a better support of model comprehension with the definition of rules as well as directives ensuring proper process model comprehension can be pursued. Moreover, knowing how a stakeholder reacts in terms of cognitive load during the comprehension of a process model allows for predictive analytics for a more focused assistance adapted to individual needs. Furthermore, tool support can be individually improved with knowledge about physiological or psychological factors (e.g., visualization) that influence process model comprehension. Finally, additional research can focus on psychological aspects pertaining to, for example, information processing, reasoning, and decision making in order to enhance our knowledge in the comprehension of process models from a neuro-centric perspective.

### 3.3. Limitations

Although the first results seem to be promising from the feasibility study, their generalization needs to be confirmed either by replication or similar studies. In particular, several limiting factors were encountered in the feasibility study that need to be discussed. First, usually, process models document complex processes of the real world. In turn, the process models used in the feasibility study were of a simple nature. As a result, complex process models require more cognitive effort for a proper comprehension, which may have led to differences in the EDA data compared to the EDA data we obtained in the feasibility study. Second, the time (i.e., t = 30 s) set for the comprehension of both process models constitutes another limitation. For the easy process model, participants could have completely comprehended the model before the time was over and, hence, the EDA signal afterwards did not correspond to the comprehension of the process model. In turn, regarding the complex process model, the time may have been set too short, which may have caused additional stress for the participants. Third, another limitation were the participants of the feasibility study. On the one hand, the sample size was small (n = 9), and only students were evaluated, which limits generalizability. Fourth, as we did not have a special lab, in which exact laboratory conditions can always prevail, the EDA measurements may have been affected due to differences in the these environmental conditions. Fifth, we did not ask the participants in the feasibility study about their physiological and psychological condition. The EDA measurement is very sensitive and, hence, different conditions (e.g., tiredness due to poor sleep) may have affected the EDA signal. Sixth, although the sample rate (i.e., 32 Hz) of the sensor EdaMove 3 is adequate for most applications, fine-grained EDA signals may have not been recorded during the measurements in the feasibility study. This is a result of a compromise to enable an adequate offline as well as online live analysis of the EDA. Seventh, in the context of EDA signal decomposition, restricted visualizations (e.g., no complete phasic SCR component illustration), missing signal corrections (e.g., no signal interpolation after filter application), and fixed parameters (e.g., >0.1 μs as the SCR amplitudes threshold; >0.05 μs is recommended in the literature [45]) also limited the data analysis. Moreover, there might be noise as well as further significant differences in the EDA data between the comprehension of the easy and complex process model, which we could not show in the feasibility study (e.g., loss of information), but which might become apparent with more accurate parameters of higher resolution (e.g., SCL low pass filter with a lower cut-off frequency than 0.1 Hz).

### 3.4. Lessons Learned

In this section, the experiences we gathered in the measurement of the EDA from the conducted research (i.e., preliminary test run and feasibility study) are summarized. In general, they constitute valuable lessons learned that will allow for optimizations of similar future studies in the context of process model comprehension:

- Baseline measurement: The baseline represents the average skin conductance level during rest and without the presence of any stimulus. Moreover, the baseline varies over time depending on various factors (i.e., physiological or psychological arousal). Therefore, it is of importance to identify a baseline level for each individual separately before the start of an EDA measurement. There are different recommendations regarding the duration of the baseline measurement, but most of the research recommend a duration between 10 and 15 min [47,51]. In our studies, we could observe that the EDA signal stabilized at a low level after about 8 minutes. In addition, the baseline measurement can be used for a more fine-grained analysis of the EDA. For example, individuals can be identified that are hyper- or hypo-responders to a stimulus. Further, during relaxation, the identification of the frequency of non-SCR (see Section 2.1) is simplified [76].
- Recording of both EDA components: The initial research only considered the phasic SCR, while the tonic SCL was not taken into account. For short-term observations (e.g., neural reaction), the SCL can be neglected. In turn, for long-term observations, both EDA components should be recorded, since both rely on different neural mechanisms. In our context, the consideration of both components allowed for the interpretation that the comprehension of process models resulted in a state of higher cognitive arousal. Finally, with the SCR, we were able to show that the comprehension of a complex process model requires more cognitive effort.
- Limit physical activity: The EDA is a very sensitive signal, and even small movements (e.g., finger movement) may cause changes in the respective signal. Depending on the accuracy of the EDA sensor device used, even contemplation may change the EDA signal. Therefore, in order to avoid such changes, we ensured that the participants in our studies did not have to perform any additional activities and could, therefore, concentrate on the comprehension of the presented process models.
- Avoid external stimuli: Similar to the activity limitation, any external stimuli (e.g., bird calls, light changes) may affect the EDA signal: several times, we could observe this effect in the test run as well (e.g., voices in the other room). Therefore, we accepted this and tried to avoid external stimuli. Hence, the recommendation is to conduct further EDA measurements in special labs (e.g., light and soundproof) to ensure a proper recording of the respective EDA components.
- Constant setting: Another important factor that needs to be considered in the measurement of the EDA is keeping a constant setting across all participants. In particular, this ensures a valid comparability of the recorded EDA signals obtained from all participants. In this context, among others, the room temperature is a critical factor that has a very strong effect on the EDA signal. A high room temperature leads to a faster increase in both EDA components (i.e., due to increased sweat production). Hence, according to existing literature, we kept the room temperature at about 22 degrees Celsius [47].
- Attention to physiological and psychological condition: Different physiological as well as psychological conditions (e.g., tiredness, digestion) affect the EDA signal. Since it is impossible to have participants with the same physiological and psychological condition, attention should be paid that EDA measurements do not directly follow strongly perceptible sensations (e.g., hunger).
- Signal decomposition: The accurate decomposition of the tonic (i.e., SCL) and phasic (i.e., SCR) component from a raw EDA signal has created a vast body of research in this context [45]. Since the two EDA components are located at sensitive frequencies, it is important to ensure that the respective methods for analysis are capable of working with fine-grained frequency ranges (e.g., >0.05 μs as the amplitude threshold for SCR detection, as recommended in the literature [77]). Therefore, the application of further robust methods for EDA analysis as proposed in the literature is recommended. However, for gaining first experiences (e.g., ambulatory setting) and in the context of the feasibility study, the sensor used (i.e., EdaMove 3) and related software (i.e., DataAnalyzer) seem to be appropriate.
- Signal transformation: Each individual has a different skin conductivity level depending on various factors (see Section 2.1). As a result, despite the similar setting, significant differences in the baseline measurement as well as SCR amplitudes may occur between individuals. For this reason, the obtained EDA results should be standardized. Established methods are log or square root transformation fostering the comparisons between individuals [47]. Moreover, physiological factors (e.g., skin thickness) as well as potential disruptive factors (e.g., non-SCR) can be disregarded with specialized transformations.
- Consideration of more factors: The measurement of the EDA allows for the interpretation about physiological as well as psychological arousal in the presence of a stimulus. For many research purposes (e.g., neural reactions on short-term events), the analysis of the EDA components is adequate. However, in our context, the sole measurement of the EDA allowed only for limited interpretation. With the tonic component SCL, we were able to show that the comprehension of process models poses demands on cognitive effort. Regarding the phasic component SCR, we observed in the feasibility study a higher number of SCR peaks during the comprehension of the complex process model, but we can only make assumptions (e.g., they may be due to decision making) regarding their appearance. Therefore, with the addition of further measurements, a better interpretation of the EDA can be assumed. For example, with sensors recording eye movements, the appearance of SCR peaks can be associated with the gaze of an individual at the time of a peak.

## 4. Conclusions and Future Work

This paper presented the first insights about the applicability of measuring the EDA in the context of process model comprehension. In the scope of the research question, the appropriateness of measuring the EDA with a specific EDA and physical activity sensor (i.e., EdaMove 3) during the comprehension of process models was evaluated. Therefore, a preliminary test run and a feasibility study were conducted. The small-scale test run was conducted to familiarize ourselves with the measurement and the analysis of the EDA and, hence, to obtain the first experiences and lessons learned. In the feasibility study, n = 9 participants needed to comprehend two differently complex BPMN 2.0 process models. The results from the feasibility study presented general variations in the tonic SCL during the comprehension of both process models. Moreover, the complex process model caused an average higher number of phasic SCR peaks compared to the easy model. Consequently, participants were confronted with a significantly higher cognitive load (i.e., level of arousal) during the comprehension of the complex process model. Hence, the number of phasic SCR peaks was identified in the feasibility study as a significant measure for the determination of distinctions in the comprehension of process models with varying model complexity. As the first work evaluating the applicability of measuring the EDA during process model comprehension, this paper makes a contribution to the respective research as well as to our existing conceptual framework (see Section 2.2), which applies measurement methods and theories from cognitive neuroscience and psychology in order to foster the comprehension of process models towards a neuro-centric perspective [57]. We demonstrated that the measurement of the EDA relying on a smart sensor can be an appropriate method, especially from a cognitive point of view, to foster our understanding of working with process models. However, the sole measurement of the EDA is not sufficient to be able to derive concrete interpretations regarding cognitive processes (e.g., decision making, reasoning) during model comprehension. Additional psychological or physiological factors have to be taken into account with the application of further technologies (e.g., heart rate sensor). Therefore, we are currently preparing another study, in which the EDA will be measured simultaneously with recorded eye movements. This will allow for a better interpretation of especially the phasic SCR peaks, as these can be correlated with eye movements (e.g., gazing at a stimulus shortly before a SCR peak). A correlation of the EDA components (i.e., tonic SCL and phasic SCR) with differently complex process model will be investigated in more detail in the same study. Moreover, although the obtained results looked promising, the conducted feasibility study was confronted with limitations regarding EDA signal decomposition (e.g., filter frequencies for EDA signal decomposition; see Section 3.3) during the analysis of the obtained EDA data. Hence, the disclosed limitations need to be addressed carefully in future work. For this reason, as these limitations are crucial in EDA signal analysis, we strive to analyze the obtained EDA data with further robust techniques and methods (e.g., SCR peak detection with an amplitude threshold of >0.05 μs) from the literature, enabling a more rigorous conception of the effects in the EDA and the respective components during the comprehension of process models [45]. Moreover, the consideration of further psychophysiological aspects (e.g., heart rate) and related technologies will be the subject of future work. Finally, such psychophysiological data are well suited in this context for further analyses, such as pattern recognition and machine learning (i.e., linear discriminant analysis) [78], as well as in order to support our understanding of working with process models. Moreover, such further analyses will allow for the identification of new insights (e.g., objective classification of stakeholders regarding the comprehension of process models based on psychophysiological measures and individual-related characteristics), enabling a better support (e.g., comprehension guidelines, tool assistance) in terms of process model comprehension in the future.

## Figures and Tables

**Table 1:** Number of modeling elements in the easy and complex process models.

Process Modeling Elements							
**Process Model**	**Activity**	**Event**	**Gateway**	**Edge**	**Pool**	**Lane**	**Total**
Easy	6	2	4	13	1	1	27
Complex	10	2	6	19	1	3	41

**Table 2:** Descriptive results for the easy process model from the feasibility study.

	Group A			Group B		
PM	Mean SCL	SCR Peak	SCR Amp	Mean SCL	SCR Peak	SCR Amp
1	11.12 (0.49)	5.00	0.21 (0.09)	5.83 (0.70)	6.00	0.75 (0.27)
2	4.83 (0.32)	7.00	0.58 (0.32)	2.82 (0.13)	4.00	0.15 (0.09)
3	3.16 (0.15)	3.00	0.18 (0.12)	4.82 (0.14)	5.00	0.23 (0.10)
4	2.79 (0.20)	6.00	0.29 (0.08)	4.87 (0.26)	4.00	0.58 (0.31)
5	2.01 (0.13)	4.00	0.12 (0.06)	–	–	–
Avg	4.78 (3.31)	5.00 (1.41)	0.28 (0.25)	4.59 (1.16)	4.75 (0.83)	0.43 (0.33)

**Note:** P = participant; M = measure; SCL = skin conductance level; SCR = skin conductance response; Amp = amplitudes energy level.

**Table 3:** Descriptive results for the complex process model from the feasibility study.

	Group A			Group B		
PM	Mean SCL	SCR Peak	SCR Amp	Mean SCL	SCR Peak	SCR Amp
1	10.44 (0.1)	4.00	0.35 (0.06)	6.46 (0.24)	9.00	0.30 (0.15)
2	4.45 (0.28)	6.00	0.56 (0.23)	2.74 (0.19)	7.00	0.26 (0.23)
3	2.24 (0.05)	8.00	0.12 (0.04)	4.90 (0.43)	10.00	0.34 (0.21)
4	3.04 (0.01)	6.00	0.24 (0.12)	2.98 (0.18)	5.00	0.29 (0.09)
5	1.83 (0.11)	8.00	0.19 (0.10)	–	–	–
All	4.40 (3.15)	6.40 (1.50)	0.29 (0.20)	4.27 (1.54)	7.75 (1.92)	0.29 (0.19)

**Note:** P = participant; M = measure; SCL = skin conductance level; SCR = skin conductance response; Amp = amplitudes energy level.

**Table 4:** Descriptive results for the SCL during baseline measurements.

Easy Process Model				Complex Process Model			
P	BM SCL	P	BM SCL	P	BM SCL	P	BM SCL
A1	11.64 (0.45)	B1	5.42 (0.78)	A1	10.60 (0.40)	B1	5.14 (0.52)
A2	4.12 (0.43)	B2	2.87 (0.22)	A2	4.10 (0.47)	B2	2.17 (0.39)
A3	3.53 (0.52)	B3	4.66 (0.69)	A3	2.51 (0.23)	B3	3.72 (0.84)
A4	2.47 (0.28)	B4	4.19 (0.67)	A4	2.64 (0.36)	B4	2.88 (0.61)
A5	2.14 (0.27)	-	-	A5	2.18 (0.53)	-	-
All	4.56 (2.74)			All	3.99 (2.56)		

**Note:** P = participant; BM = baseline measurement; SCL = skin conductance level.

**Table 5:** Descriptive results for the number of non-SCR peaks during baseline measurements.

Easy Process Model				Complex Process Model			
P	BM SCR	P	BM SCR	P	BM SCR	P	BM SCR
A1	5.05 (0.74)	B1	5.75 (0.94)	A1	3.80 (0.75)	B1	4.55 (1.40)
A2	4.15 (1.01)	B2	4.20 (0.79)	A2	4.15 (0.96)	B2	4.65 (1.01)
A3	2.55 (0.74)	B3	4.75 (0.83)	A3	5.05 (1.24)	B3	4.50 (1.86)
A4	5.30 (1.68)	B4	3.65 (0.79)	A4	4.60 (1.59)	B4	4.70 (1.42)
A5	3.95 (1.16)	-	-	A5	4.90 (1.30)	-	-
All	4.39 (0.95)			All	4.55 (1.30)		

**Note:** P = Participant; BM = baseline measurement; SCR = number of non-skin conductance response peaks.

**Table 6:** Descriptive results for the SCR amplitudes energy level during baseline measurements.

Easy Process Model				Complex Process Model			
P	BM Amp	P	BM Amp	P	BM Amp	P	BM Amp
A1	0.22 (0.09)	B1	0.47 (0.19)	A1	0.33 (0.14)	B1	0.24 (0.09)
A2	0.55 (0.18)	B2	0.20 (0.05)	A2	0.60 (0.18)	B2	0.26 (0.15)
A3	0.16 (0.04)	B3	0.23 (0.12)	A3	0.18 (0.07)	B3	0.31 (0.12)
A4	0.26 (0.08)	B4	0.53 (0.17)	A4	0.24 (0.08)	B4	0.29 (0.12)
A5	0.15 (0.05)	-	-	A5	0.20 (0.07)	-	-
All	0.31 (0.11)			All	0.27 (0.11)		

**Note:** P = participant; BM = baseline measurement; Amp = amplitudes energy level.

**Table 7:** Descriptive results for the easy and complex process model.

PM	PM SCL	SCR Peak	SCR Amp
Easy	4.70 (2.56)	4.89 (1.20)	0.37 (0.30)
Complex	4.34 (2.57)	7.00 (1.83)	0.29 (0.19)

**Note:** PM = process model; SCL = skin conductance level; SCR = skin conductance response; Amp = amplitudes energy level.

## Abbreviations

The following abbreviations are used in this manuscript:

BPMN	Business Process Model and Notation
DC	Direct current
EDA	Electrodermal activity
EEG	Electroencephalography
GAPED	Geneva Affective Picture Database
SCL	Skin conductance level
SCR	Skin conductance response
